# Supplementary material for: A computational framework for modelling infectious disease policy based on age and household structure with applications to the COVID-19 pandemic
Source: PLoS Comput Biol. 2022 Sep 6;18(9):e1010390. doi: 10.1371/journal.pcbi.1010390 (PMC9481179; doi:10.1371/journal.pcbi.1010390)
Supplement: S1 Appendix — Fig A in S1 Appendix. Current class structure of the software. Fig B in S1 Appendix. Convergence of model from initial conditions to exponential growth regime. Table A in S1 Appendix. Wall times for case study calculations. (PDF) [file pcbi.1010390.s001.pdf]

Appendix to *A computational framework for modelling infectious disease policy based on age and household structure with applications to the COVID-19 pandemic*

Joe Hilton[1,2,*], Heather Riley[3], Lorenzo Pellis[3,4], Rabia Aziza[1,2], Sam Brand[1,2,5], Ivy K. Kombe[5], John Ojal[5,6], Andrea Parisi[1,2], Matt J. Keeling[1,2,7], D. James Nokes[1,2,5], Robert Manson-Sawko[8], and Thomas House[3,4,8]

[1]School of Life Sciences, University of Warwick, Coventry, UK

[2]Zeeman Institue (SBIDER), University of Warwick, Coventry, UK

[3]Department of Mathematics, University of Manchester, Manchester, UK

[4]The Alan Turing Institute for Data Science and Artificial Intelligence, London, UK

[5]Kenya Medical Research Institute – Wellcome Trust Research Programme, Kilifi, Kenya

[6]Department of Infectious Disease Epidemiology, London School of Hygiene & Tropical Medicine, London, UK

[7]Mathematics Institute, University of Warwick, Coventry, UK

[8]IBM Research Europe, Hartree Centre, Daresbury, UK

[*]Corresponding Author: joe.hilton@warwick.ac.uk

1 Computational implementation

Our household-structured infectious disease model has been released as an open-source software package [1] under Apache License (version 2). It has been implemented in Python following guidelines for small scientific projects [2].

The core of the implementation is stored in the `model/` directory as a set of functions and classes automating the construction of data structures for household populations and rate equations.

The current class structure is presented in Figure a. The `RateEquations` class is implemented as a functor. An instance of this class can be passed as an input to an ODE integrator such as `solve_ivp`. The `RateEquations` class depends on the `HouseholdPopulation`. Instances of the latter need to be constructed beforehand. The population class constructs the internal transmission matrix $\boldsymbol{Q}_{\text{int}}$. Both classes can be inherited from to create specialised instantiations of household population objects or rate equations e.g. `SEPIRRateEquations` or `SEPIRInput`.

Examples of the analyses are given in the `examples` directory, with each example contained within a subdirectory. In each subdirectory the following file structure is maintained:

- `common.py` a module containing any common classes or functions for this specific example,
- `plot_sweep_results.py` file to produce figures from intermediate output files
- `parameter_sweep.py` parameter sweep files, possibly parallel sweeps.

The inputs to the code are passed as key-value pairs and are bundled into Python dictionaries. Example dictionaries have been collected in `model/specs.py` and serve as default settings for parametric sweeps. Some fields point to files containing demographic input data e.g. data from UK census. These files have been placed in `inputs/` directory and are stored in mixed formats e.g. CSV and Excel® format.

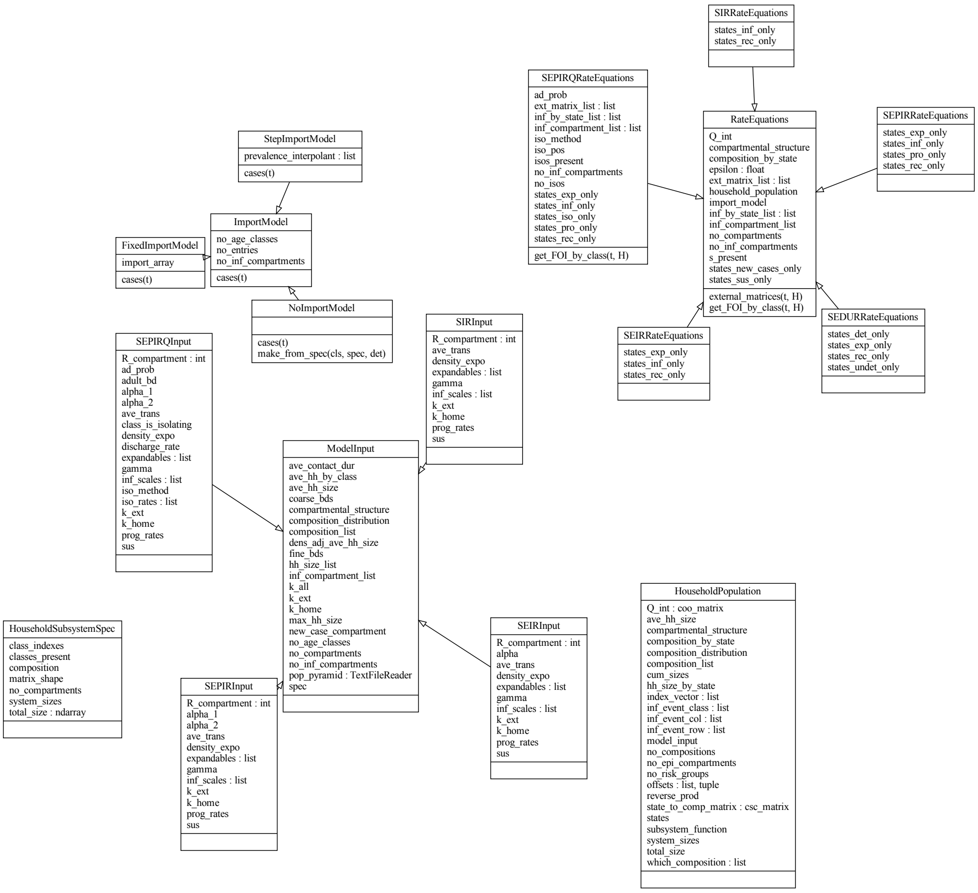

Figure A: Current class structure of the software.

Table a presents a result of a benchmark that was carried out for the examples in the repository [1]. All experiments are parametric sweeps and we used default ranges that were fixed in the code. Each simulation in the sweep is run in serial, but the sweep is parallelised using `multiprocessing` package, which effectively creates separate processes running concurrently. Because of this threading employed by underlying numerical packages, the thread count was limited to one in order to avoid oversubscription of available hardware resources.

To demonstrate our model framework's ability to rapidly calibrate to epidemic doubling time or growth rate estimates, we use the script `examples/between_hh_fitting/repeat_mixing_fits.py` to perform repeated calibrations. We initialise our model using the baseline UK-like parameters used for our analysis of transmission controls at the within- and between-household level in section 3.1 of the main paper, with no controls implemented, and use the Euler-Lotka method derived in section 2.4 to calibrate the model to growth rates drawn uniformly at random from the interval $[-0.1, 0.5]$. Because the matrix $\boldsymbol{Q}_{\text{int}} - r\boldsymbol{I}$ is close to singular for values of r which are close to the eigenvalues of $\boldsymbol{Q}_{\text{int}}$ (which includes zero since $\boldsymbol{Q}_{\text{int}}$ is a stochastic matrix), the calibration

method will fail for growth rates close to these values. We attempted to calibrate to 1,000 growth rates, of which 9,983 were successful, with the remainder failing because the chosen growth rate was close to zero. The values which caused the calibration to fail were all on the order of 10^{-4} or smaller, suggesting that growth rates on the order of one tenth of a percentage percentage point or larger should be allow for successful calibration. Our 1,000 attempts took 1,315 seconds on an Intel Skylake processor. These attempts were run in serial, so that the mean time taken for a single attempt was on the order of one second.

**Experiment**	**Wall time** [s]
`external_isolation`	1141.8
`long_term_bubbles`	3201.9
`mixing_sweep`	94.9
`temp_bubbles`	1366.5

Table A: The results obtained with `multiprocessing` package or sweep parallelism and by limiting multithreading of underlying mathematical libraries. The experiments were run on Intel Skylake E5-2697A v4 2.60GHz processor with 2 sockets and 16 physical cores each with hyper-threading enabled.

2 Impact of background immunity on early growth

In this section we explore the impact of background immunity levels on the early growth of the epidemic by replicating the analysis which we used to generate Figure 1 of the main paper over a range of initial conditions with differing background immunities. In Figure b we plot the early growth in cases for epidemics with starting conditions calibrated to a doubling time of 3 days (growth rate $r = \log(2)/3$) with initial prevalence of 10^{-2} and background immunity ranging from zero up to 10^{-1}, alongside an exponential curve with rate r calibrated in each case to the simulated prevalence at time $t = 7$ days. In each case there is a close match between the simulated growth in cases and the exponential curve, suggesting that even for relatively high background immunities (up to 10%, the highest level which appears in any of our case studies) we are justified in using the Euler-Lotka method to calibrate our transmission rates and initial conditions to a doubling time or exponential growth rate.

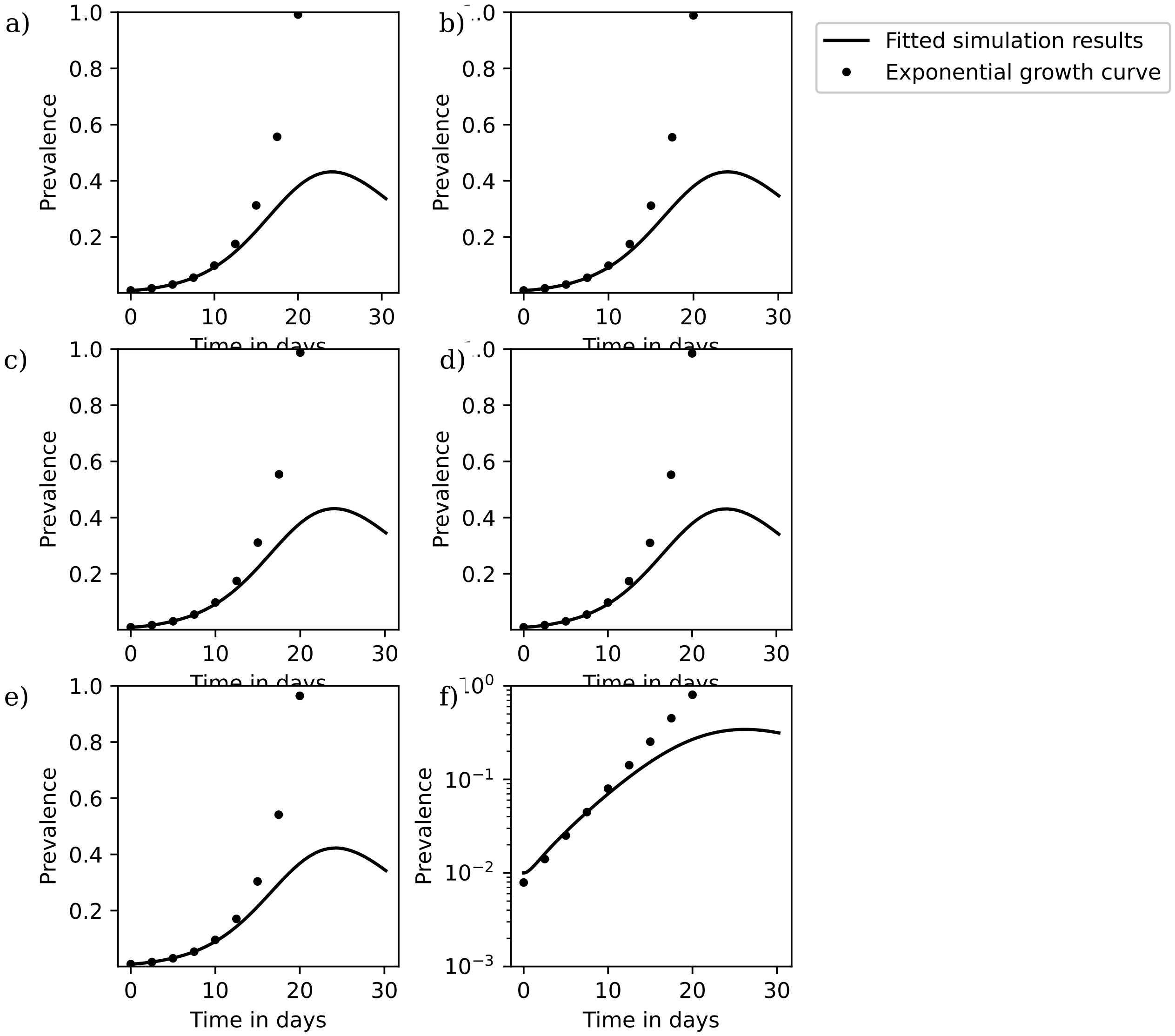

Figure B: Convergence of model from initial conditions to exponential growth regime. The model is simulated from initial conditions with starting prevalence 10^{-2} and a background immunity of a) 0, b) 10^{-5}, c) 10^{-4}, d) 10^{-3}, e) 10^{-2}, and d) 10^{-1}. The exponential curve is calibrated to the prevalence 7 days from the start of the simulation.

References

[1] Hilton J, Manson-Sawko R, House T, Riley H. hh-npi-modelling; 2021. Available from: `https://github.com/JBHilton/hh-npi-modelling`.

[2] Rokem R, Vanderplas J, Halchenko Y. Shablona: a template for small scientific Python projects; 2021. Available from: `https://github.com/uwescience/shablona`.
