# Supplementary material for: A computational framework for modelling infectious disease policy based on age and household structure with applications to the COVID-19 pandemic
Source: PLoS Comput Biol. 2022 Sep 6;18(9):e1010390. doi: 10.1371/journal.pcbi.1010390 (PMC9481179; doi:10.1371/journal.pcbi.1010390)
Supplement: Attachment — Submitted filename: review.pdf [file pcbi.1010390.s002.pdf]

A computational framework for modelling infectious disease policy based on age and household structure with applications to the COVID-19 pandemic

This manuscript presents a new modelling framework for estimating the impact of NPIs in disease control. The key novelties of the framework are (i) that it combines both age/risk structure AND household structure (many existing frameworks focus on one or other of these); and (ii) that it is formulated as systems of ODEs, making the model more tractable to general analysis (many existing frameworks that do incorporate multiple dimensions of structure are individual-based and hence less amenable to analysis). The capabilities of the framework are demonstrated via a series of case studies based on the UK experience of COVID-19.

The framework presented is novel, elegant, likely to be of interest to other modellers, and genuinely distinctive from the plethora of other COVID-19 models out there. The manuscript is enhanced by the open availability of code (which is accessible, well-structured and commented).

There are however, substantial opportunities for improving how the model framework is presented in the manuscript. Hence, most of my comments below are focused on the introduction, framing and discussion of the case studies, and general discussion of the framework itself in the context of the broader field of infectious disease models generally (and COVID-19 models).

To summarise my main concerns very briefly (but see below for detail):

- The introduction could be more focused.
- The case studies could be better motivated in terms of both the decision problem they addressed, and the specific model capability they demonstrate.
- There could be greater reflection on the strengths and limitations of the framework presented, particularly with respect to those of other modelling approaches.

Abstract

The concept of “age/risk structure” (line 21) feels a bit “abstract” for an abstract. Given that “age” is used the title, I suggest being consistent here. You elaborate on the broader interpretation later.

Introduction

The introduction is fine, but wanders a bit before getting to the point, touching on a range of interesting problems: variation in (disease) outcomes are mentioned (line 64), but these aren’t mentioned again in the manuscript; similarly, impact of household isolation on TTI policies is mentioned (line 67), again for the only time in the manuscript; the challenge of “rapidly changing situations” (referring to just the disease dynamics, or social behaviour and policy settings?) is mentioned (line 74), but not really discussed further. I suggest focusing the background on the problem that you *do* address in this manuscript, which – somewhat curiously – is only hinted at obliquely on line 76 (“attempting to predict more complex outcomes”, the precise nature of which remain unspecified). Related, NPIs are referred to in the abstract and author summary, but not in the Introduction (or at least only indirectly, in terms of “potentially infectious individuals isolating within households” (line 66). Incidentally, I would disagree that NPIs “typically act on the household

level" (line 38) given that NPIs can incorporate policies as diverse as environmental cleaning, mask wearing, and coughing into your elbow.

The use of mathematically tractable formulations is stated as a benefit (line 80) (and I don't disagree); however, the evidence provided – particularly in the context of COVID-19 – is pretty thin. Given that individual-based stochastic simulations *have* been heavily relied upon for policy formulation in the UK and elsewhere, could you motivate this work by highlighting some of the specific limitations of those approaches, particularly in a policy context (without getting too political). A brief discussion of this point would greatly strengthen the motivation for the current work.

From a methodological perspective, it isn't clear (in the Introduction) how the present model extends on ref [20]. Is it just the application to the four specific policy analyses? Or was further methodological development required?

The summary of work presented ("derive two analytical results", etc; line 94) could be better contextualised in the specific challenges that motivated the work. (And "observational studies" of what, by the way? There are several points such as this where it feels that statements could benefit from being made more concrete). It would be helpful to introduce a bit more narrative structure about how the various components of work link together. Is it possible to motivate the choice of interventions? For example, do they help illustrate different capabilities of the model?

Minor: 66: "At the same time, …" : as what?

Methods

151: To clarify, I'm not sure I understood the parenthetical comment about the possibility of time-dependent external imports: do you mean situations where the "evolving infectious population" might be larger than the "entire population of households" that is being explicitly modelled?

169: I feel the "prodromal" is a sufficiently unusual term in the context of infectious disease modelling (even if the concept is now very familiar) that it warrants definition (at first usage, the definition around line 22, where line numbers vanish momentarily, is fine).

188: I'd forgotten what SITP was by this point; perhaps spell out again in this section.

237: "…when population immunity is at low levels." I suggest "when few people in the population are immune." (if this is what you mean) The current phrasing could encompass, eg, waned/partial immunity across the population which is not what is intended.

Page 13 (bottom): "Pollett and Stefanov" needs a citation (twice).

303: "The dataset includes household compositions which are large comparatively rare." (missing "and"?)

310: "OOHI": spell out in full again for this section.

Figure 2: I'm not sure that this adds much to the manuscript – consider moving to SI (or code documentation).

313: It may be helpful to define the term "shielding" in this context (as with "bubbling", I think more heavily used in the UK than elsewhere, so not generally interpretable).

Question: How does the model account for the observed overdispersion of COVID-19? (eg, https://dx.doi.org/10.12688%2Fwellcomeopenres.15842.3 and other studies) Is this implicitly captured by the household size distributions and mixing matrices?

Results and discussion

410: Can you provide examples of measures to reduce within-household transmission? I can think of self-isolation for symptomatic cases; however, this (reactive) measure feels like it would have different implications to a simple reduction in \beta_int (it would not be effective for household members in P, for a start). More generally, assuming that this is the first of the "case studies" used to demonstrate the utility of your framework, it would be helpful to provide a bit more context: what was the decision that this modelling was conducted to inform? How was this translated into the specific scenarios to explore? How then do the results shown in Figures 3 & 4 inform this decision. The current interpretation of results describes a series of observations, but would be strengthened by greater focus on the why these observations matter in the context of a specific decision. (nb: this applies to the later case studies as well)

412: "external mixing intensity": please map to the relevant parameter/equation from the methods section.

418: "solve the self-consistent equations": please refer to equation numbers.

420: The observation that reducing between household mixing has a greater impact on growth rate feels intuitive – can this be related to existing results in the literature?

481: I'm assuming that the baseline value of \beta_int is used here? (ie, the values along the x-axes in Figure 3, where % reduction is 0.0, are equivalent to the values along the x-axes in Figure 5, where the % uptake is 0.0?). It may be helpful to state this.

Figure 6: viridis is beautiful, but I find these gradients quite difficult to interpret. Would it be straightforward to plot contour lines corresponding to peak prevalence at .5% intervals (as used in the legend)?

564: "callibrated"

564: This paragraph has lots of pretty vague terms: "comparatively large", "moderately fast detection", "moderately high isolation probability". I appreciate that the precise values aren't really the point here, but it has the unfortunate side-effect of making the final paragraph of the section come of as a bit wishy-washy. See comment above about providing a bit more information on the decision context in which these case studies were embedded. I suspect it could make for a stronger conclusion to the section.

Question: Am I correct in my assumption that the policies modelled (bubbles, etc) implicitly assume full compliance? Presumably the impact of bubbling might be greater if people took advantage of such a policy to form a bubble with one household on one day and different household on a subsequent day. I subsequently see that this is perhaps captured by one of the short-term bubbling scenarios, although only over a limited time period – would it make sense to interpret these results in terms of "imperfect compliance with long-term bubbling"?

Suggestion: While not necessarily the most enthralling of results, given that one of the stated strengths of the modelling approach is its tractability in comparison to more detailed individual-based simulations, it may be useful to provide some example statistics on model runtimes.

Suggestion: The methods section is very long, and relies on concepts that are only described more comprehensively in the results (eg, the context under which "social bubble" policies were proposed). I wonder if the paper would read more naturally if the methods for each particular case study were embedded with the background context, results and discussion for that case study, with the "main" methods reserved for the general model framework alone. At the very least, this would reduce the level of flipping backwards and forwards that is required to interpret the case studies, and perhaps allow for a more natural flow.

573: Conclusions – the conclusions summarise the insights derived in each of the case studies. However, these aren't really the primary contribution of this study. In a sense, they're not even particularly interesting anymore, as the specific questions of interest move on with each twist of COVID epidemiology and political whim. I think there is opportunity here though to reflect more deeply on how the very nice model framework presented here was perhaps better suited to addressing this class of question than other approaches.

In addition, the traditional section on "model limitations" is absent here, which I think is a shame, as while the availability of code will hopefully enable others to make use of the framework, it would be helpful to allow them to go into it "with eyes open", alert to the specific data requirements, constraints, etc. that are a part of any model (eg, implicit assumptions about compliance, omission of focused mixing environments other than the household, etc).
