# Supplementary material for: A computational framework for modelling infectious disease policy based on age and household structure with applications to the COVID-19 pandemic
Source: PLoS Comput Biol. 2022 Sep 6;18(9):e1010390. doi: 10.1371/journal.pcbi.1010390 (PMC9481179; doi:10.1371/journal.pcbi.1010390)
Supplement: Attachment — Submitted filename: HH paper response.pdf [file pcbi.1010390.s003.pdf]

Dear Editors

We would like to thank the reviewers for their insightful comments. These have been invaluable to us and we feel that they have dramatically improved the quality of our paper. Our detailed response to these comments are given below.

Best wishes
Joe Hilton and co-authors

Response to Reviewer #1

To summarise my main concerns very briefly (but see below for detail):

- *- The introduction could be more focused.*
  We have substantially revised the introduction section.
- *- The case studies could be better motivated in terms of both the decision problem they addressed, and the specific model capability they demonstrate.*
  We have expanded the introductory paragraphs of each case study, with reference to relevant UK government documents were possible. We have added an explanation of the model capacities demonstrated by the case studies to our Introduction section (lines 136-149).
- *- There could be greater reflection on the strengths and limitations of the framework presented, particularly with respect to those of other modelling approaches.*
  We have added a discussion of the model's limitations to the papers' discussion section.

Abstract

The concept of "age/risk structure" (line 21) feels a bit "abstract" for an abstract. Given that "age" is used the title, I suggest being consistent here. You elaborate on the broader interpretation later.

We have replaced both mentions of "age/risk structure" in the abstract with mentions of age structure.

Introduction

The introduction is fine, but wanders a bit before getting to the point, touching on a range of interesting problems: variation in (disease) outcomes are mentioned (line 64), but these aren't mentioned again in the manuscript; similarly, impact of household isolation on TTI policies is mentioned (line 67), again for the only time in the manuscript; the challenge of "rapidly changing situations" (referring to just the disease dynamics, or social behaviour and policy settings?) is mentioned (line 74), but not really discussed

*further. I suggest focusing the background on the problem that you *do* address in this manuscript, which – somewhat curiously – is only hinted at obliquely on line 76 ("attempting to predict more complex outcomes", the precise nature of which remain unspecified). Related, NPIs are referred to in the abstract and author summary, but not in the Introduction (or at least only indirectly, in terms of "potentially infectious individuals isolating within households" (line 66). Incidentally, I would disagree that NPIs "typically act on the household level" (line 38) given that NPIs can incorporate policies as diverse as environmental cleaning, mask wearing, and coughing into your elbow.*

We have substantially revised the Introduction section; in particular, we now motivate the use of household and age structure in consideration of NPIs, while making clear that these are very diverse, as well as other changes as suggested.

*The use of mathematically tractable formulations is stated as a benefit (line 80) (and I don't disagree); however, the evidence provided – particularly in the context of COVID-19 – is pretty thin. Given that individual-based stochastic simulations *have* been heavily relied upon for policy formulation in the UK and elsewhere, could you motivate this work by highlighting some of the specific limitations of those approaches, particularly in a policy context (without getting too political). A brief discussion of this point would greatly strengthen the motivation for the current work.*

The main benefit of the mathematically tractable formulation of our model is that it allows for fast calibration to estimates of doubling time or exponential growth rate. In the supplement attached to our revised manuscript we provide benchmarking statistics quantifying this speed. We also note in the Introduction that in studies focused on issues such as vaccination, there are expected to be other analytical results available (lines 123-125).

From a methodological perspective, it isn't clear (in the Introduction) how the present model extends on ref [20]. Is it just the application to the four specific policy analyses? Or was further methodological development required?

The model in ref [20] was only able to calculate early growth rates and long-term equilibria of an epidemic, and could not be used to perform direct "real time" simulations. The focus on direct simulation by numerical integration of the self-consistent equations is the main methodological advance of the present study relative to the one in ref [20]. We have added a brief discussion of this to lines 103-106 of the manuscript.

The summary of work presented ("derive two analytical results", etc; line 94) could be better contextualised in the specific challenges that motivated the work. (And "observational studies" of what, by the way? There are several points such as this

where it feels that statements could benefit from being made more concrete). It would be helpful to introduce a bit more narrative structure about how the various components of work link together. Is it possible to motivate the choice of interventions? For example, do they help illustrate different capabilities of the model?
We have revised the reference to our analytical results with a more detailed summary (lines 110-116).
We have also expanded the summary of our policy analyses with closer reference to the model capacities illustrated (lines 136-1492).

Minor: 66: "At the same time, ..." : as what?
This sentence has been revised, so that it now begins as follows: "The UK's pandemic response has focused on…"

Methods
151: To clarify, I'm not sure I understood the parenthetical comment about the possibility of time- dependent external imports: do you mean situations where the "evolving infectious population" might be larger than the "entire population of households" that is being explicitly modelled?
As you intuit, we were thinking here of scenarios where our households model could be coupled to a growth curve describing the infectious disease dynamics of a larger population. While our code has the capacity to model these scenarios, we do not consider them elsewhere in the paper, and so we have removed this comment.

169: I feel the "prodromal" is a sufficiently unusual term in the context of infectious disease modelling (even if the concept is now very familiar) that it warrants definition (at first usage, the definition around line 22, where line numbers vanish momentarily, is fine).
A definition has been added at the start of Section 2.2, where we introduce the SEIR and SEPIR models. (lines 225-227)

188: I'd forgotten what SITP was by this point; perhaps spell out again in this section.
We have spelt out the acronym in full here. (Line 287).

237: "...when population immunity is at low levels." I suggest "when few people in the population are immune." (if this is what you mean) The current phrasing could encompass, eg, waned/partial immunity across the population which is not what is intended.
We have changed the wording in line with the reviewer's suggestion.

Page 13 (bottom): "Pollett and Stefanov" needs a citation (twice).

We have added a second citation.

303: "The dataset includes household compositions which are large comparatively rare." (missing "and"?)
This has been corrected.

310: "OOHI": spell out in full again for this section.
This has been spelt out in full.

Figure 2: I'm not sure that this adds much to the manuscript – consider moving to SI (or code documentation).
We have moved this figure, along with the accompanying explanation of the computational implementation, to the Supplementary Material.

313: It may be helpful to define the term "shielding" in this context (as with "bubbling", I think more heavily used in the UK than elsewhere, so not generally interpretable).
We have added definitions of "shielding" and "bubbling", with references to UK government documents, on lines 490-492 and 602-604 respectively.

***Question*: How does the model account for the observed overdispersion of COVID-19? (eg, https://dx.doi.org/10.12688%2Fwellcomeopenres.15842.3 and other studies) Is this implicitly captured by the household size distributions and mixing matrices?**

We have now added a paragraph to the Discussion section mentioning that there is some implicit overdispersal in household models, but that there are possibilities to extend the level of this still further that could be considered in future work (lines 878-883).

Results and discussion
410: Can you provide examples of measures to reduce within-household transmission? I can think of self-isolation for symptomatic cases; however, this (reactive) measure feels like it would have different implications to a simple reduction in \beta_int (it would not be effective for household members in P, for a start). More generally, assuming that this is the first of the "case studies" used to demonstrate the utility of your framework, it would be helpful to provide a bit more context: what was the decision that this modelling was conducted to inform? How was this translated into the specific scenarios to explore? How then do the results shown in Figures 3 & 4 inform this decision. The

current interpretation of results describes a series of observations, but would be strengthened by greater focus on the why these observations matter in the context of a specific decision. (nb: this applies to the later case studies as well)
We have added references to policy documents from the UK where within-household control measures were discussed, as well as randomised control trials from before the pandemic which looked at within-household controls on influenza transmission. We have emphasised that our goal here is to assess the potential impact of within-household transmission controls on population-level spread of infection, rather than the impact of any specific measure on within-household transmission. (Lines 521-535)

412: "external mixing intensity": please map to the relevant parameter/equation from the methods section.
We have added the notation for the external mixing intensity here as well as a reference to the formula we derive for it in the section on the Euler-Lotka equation. (Line 536-537)

418: "solve the self-consistent equations": please refer to equation numbers.
We have now added the appropriate equation number reference. (Line 542)

420: The observation that reducing between household mixing has a greater impact on growth rate feels intuitive – can this be related to existing results in the literature?
This observation is very consistent with the findings of Ball *et al's* classic theoretical study on "epidemics with two layers of mixing", where they demonstrate that R0 can not be driven below zero unless the number of between-household infections generated by each case is below 1. We have added a discussion of this result and how it relates to our findings on within-household control measures on 548-558.

481: I'm assuming that the baseline value of \beta_int is used here? (ie, the values along the x-axes in Figure 3, where % reduction is 0.0, are equivalent to the values along the x-axes in Figure 5, where the % uptake is 0.0?). It may be helpful to state this.
We have added the following clarification on lines 637-638:
"The transmission rate over within- household contacts, β_int is left fixed at the value obtained by fitting to the SITP, listed in Table 1. This reflects the fact that our primary interest here is specifically in modelling the potential detrimental impact of support bubble formation on measures to reduce between-household transmission."

Figure 6: viridis is beautiful, but I find these gradients quite difficult to interpret. Would it be straightforward to plot contour lines corresponding to peak prevalence at .5% intervals (as used in the legend)?
We have added contour lines to all of the heatmap plots in our paper.

564: “callibrated"
This has been corrected.

564: This paragraph has lots of pretty vague terms: “comparatively large”, “moderately fast detection”, “moderately high isolation probability”. I appreciate that the precise values aren’t really the point here, but it has the unfortunate side-effect of making the final paragraph of the section come of as a bit wishy-washy. See comment above about providing a bit more information on the decision context in which these case studies were embedded. I suspect it could make for a stronger conclusion to the section.
We have added a more specific discussion of the policy context for this case study at the beginning of this section (lines 787-789). To make our results more concrete, we have replotted the peak and cumulative prevalences in the vulnerable class as percentages of the baselines which would be attained if no OOHI policy were implemented. We have revised the paragraph with more specific reference to the levels of reduction which could be achieved and how they balance against the number of people entering isolation (lines 822-844).

Question: Am I correct in my assumption that the policies modelled (bubbles, etc) implicitly assume full compliance? Presumably the impact of bubbling might be greater if people took advantage of such a policy to form a bubble with one household on one day and different household on a subsequent day. I subsequently see that this is perhaps captured by one of the short-term bubbling scenarios, although only over a limited time period – would it make sense to interpret these results in terms of “imperfect compliance with long-term bubbling”?
Our model does not explicitly account for compliance, and our analyses of both bubble policies assume that individuals will follow regulations to the letter. We have added a discussion of how our model could be extended to model questions around compliance to the Discussion section of the paper (lines 906-921).

Suggestion: While not necessarily the most enthralling of results, given that one of the stated strengths of the modelling approach is its tractability in comparison to more detailed individual- based simulations, it may be useful to provide some example statistics on model runtimes.
We have added a supplement to the paper which includes wall times for each of the analyses in the results section as well as for repeated calibration to growth rate estimates.

Suggestion: The methods section is very long, and relies on concepts that are only described more comprehensively in the results (eg, the context under which “social

bubble" policies were proposed). I wonder if the paper would read more naturally if the methods for each particular case study were embedded with the background context, results and discussion for that case study, with the "main" methods reserved for the general model framework alone. At the very least, this would reduce the level of flipping backwards and forwards that is required to interpret the case studies, and perhaps allow for a more natural flow.

We have rearranged the Methods and Results sections of the manuscript in line with these suggestions.

573: Conclusions – the conclusions summarise the insights derived in each of the case studies. However, these aren't really the primary contribution of this study. In a sense, they're not even particularly interesting anymore, as the specific questions of interest move on with each twist of COVID epidemiology and political whim. I think there is opportunity here though to reflect more deeply on how the very nice model framework presented here was perhaps better suited to addressing this class of question than other approaches.

In addition, the traditional section on "model limitations" is absent here, which I think is a shame, as while the availability of code will hopefully enable others to make use of the framework, it would be helpful to allow them to go into it "with eyes open", alert to the specific data requirements, constraints, etc. that are a part of any model (eg, implicit assumptions about compliance, omission of focused mixing environments other than the household, etc).

We have added a discussion of model limitations at the end of our Discussion section (lines 872-926).

Response to Reviewer #2

General comments

1) Please provide more consistent sub-headings and descriptions of the four policy analyses throughout the manuscript so the reader can more easily comprehend your work.

For example, the introduction lists the following policy analyses:

** Control of within- and between-household mixing through NPIs*
** Formation of support bubbles during lockdown periods*
** Out-of-household isolation*
** Temporary relaxation of NPIs during holiday periods*

The methods section lists the following “complex” interventions:

**Long-term household bubbling - which is the “support bubbles”*
**Short-term household bubbles - which is the temporary relaxation of NPIs during holiday periods*
**Out-of-household isolation*

The results and discussion section contains the following sub-sections:
**Impact of NPIs at the within- and between-household level*
**Long-term social bubbles*
**Short-term social bubbles*
**Out of household isolation*

In line with a suggestion from Reviewer 1, we have merged the case study sections of the Methods and Results into a single set of case studies under the following titles:
* Impact of transmission controls at the within- and between-household level
* Lockdown support bubbles
* Temporary relaxations of NPIs
* Out-of-household isolation

The description of the first “policy analysis” is particularly confusing since all of the interventions explored in the study are NPIs. I suggest coming up with a more specific title for this analysis.
Here we have replaced “NPI’s” with the more specific term “transmission controls”.

2) I’m not (yet) convinced of the utility of the “between-household” and “within-household” transmission controls analysis. It’s not clear to me how specific policies can be placed into these two categories. I wonder if the analysis would be more useful for policymakers if it were re-framed as “case targeted” and “community-wide” controls, with the careful consideration of within and between household transmission dynamics that your modelling framework allows. Case isolation and contact tracing and quarantine would be examples of case targeted controls and workplace closures and lockdowns would be examples of community-wide controls. Furthermore, have you considered that some community wide NPIs such as lockdowns may decrease between household transmission and increase within household transmission? And depending on what you consider a “within-“ versus “between-household” control, I’m not sure how helpful the following conclusion is “...suggests that within-household measures likely to have a limited impact due to the high proportion of non-household transmissions which

characterise the spread of COVID-19" - what specific measures are therefore likely to have a limited impact?

Our analysis of within-household transmission controls is intended to assess the possible impact of within-household transmission controls on population-level transmission of infection. Within-household measures were considered by the UK government in early 2021, as evinced by this document which includes examples of potential measures: https://www.gov.uk/government/publications/emgspi-bspi-m-reducing-within-and-between-household-transmission-in-light-of-new-variant-sars-cov-2-14-january-2021. In this document the potential government action targeting within-household transmission focuses on an information campaign designed to encourage uptake of by individuals of measures including hand hygiene, mask-wearing in the home, and better ventilation practice. The distinction between case-targeted and community-wide measures does not quite align with our distinction between measures targeting within- and between-household transmission, since, for example, both case isolation and lockdown measures act by reducing infection between households rather than within them, but case isolation is a case-targeted control and lockdown measures are community-wide. Rather than directly comparing measures focusing at the two levels, our analysis is intended as an assessment of the utility of within-household measures as a whole, since it is not obvious a priori what the impact of a given reduction in within-household transmission will be in terms of population-level burden of disease, particularly if we expect to see multiple imports of transmission into each household. To address these points, we have expanded the opening to this study with references to the UK government document linked above, and pre-pandemic case studies on the impact of hand hygiene and within-household masking measures.

3) Please add further reflection of other relevant studies in the introduction and/or discussion. For example, the approach of other model-based NPI policy analyses.

We have added a brief account of the methods typically used in similar analyses and the types of policies which have been addressed by modeling, with an emphasis on the novelty of our deterministic age-and-household-structured approach (lines 126-135).

4) Please add a discussion of the study limitations.

We have added this discussion to the Discussion section which closes our revised manuscript (lines 872-926).

5) Please consider including a schematic overview at the start of the methods section showing the links between the model components, data inputs and policy analyses.

We agree that such a schematic is a good idea, and attempted to implement one, however we were not satisfied that there was sufficient commonality in the pipelines

involved in each sub-analysis - at least partially due to the extremely fast pace with which data availability and policy questions changed during the COVID-19 pandemic - to do this in a way that was ultimately satisfactory.

Specific comments

Methods

I'm not clear on the link(s) between the risk classes, within-household transmission and the parameterisation to estimates of SITP. How does susceptibility and infectiousness vary by risk class? Does the demographic composition of a household (and so risk class of individuals in a household) impact the SITP? How do these assumptions or estimates relate to other studies of age-specific susceptibility and transmissibility (e.g. Davies et al https://www.nature.com/articles/s41591-020-0962-9).

Our formula for the SITP is defined in terms of an "average" contact between members of the same household; the duration term T_int can be interpreted as the expected daily duration of contact between a randomly chosen individual and a randomly chosen member of their own household, where the expectation is taken over the age class of the susceptible individual and the age class of their contact. This average is given by the leading eigenvalue of the matrix in Equation 6, following the same reasoning given in Keeling and Rohani's textbook. We have added a reference to this textbook on LINE. The averaging over the age classes of the two individuals does not explicitly take household demographic structure into account, but the contact matrix K_int will implicitly reflect an underlying contact structure since recorded within-household contacts will be longer and more frequent between pairs of age classes which typically share households.

We have added some references to studies on age-specific susceptibility and transmissibility to the Introduction section (lines 92-93). Our model assumes uniform susceptibility and transmissibility across risk classes, and identical disease progression parameters. This choice was made when we began to develop the model in the early stages of the pandemic, in light of the mixed evidence on age-specific susceptibility and transmissibility at that time and the inherent difficulties in disentangling these two heterogeneities from one another.

Line 183. What is the structure of the within-household social contact matrix? Is it informed by data?

The within-household contact matrix is taken from the estimates published by Prem *et al.* (see https://journals.plos.org/ploscompbiol/article?id=10.1371/journal.pcbi.1005697), as is the between-household contact matrix. We have specified this on line 256.

Since you describe two ways in which the models are calibrated to observational studies (SITP and use of Euler-Lotka equation), please consider adding a sub-heading (around line 187) for the use of SITP estimates for parameterising within-household transmission (at the same heading level as the Euler-Lotka).

We have added a subsection heading “Estimation of within-household mixing parameters” to this section. To keep the ordering of subsections and sub-subsections rational, we have moved this subsection so that it now follows the discussion of the SEPIR and SEIR compartmental structures, and precedes the subsection on the Euler-Lotka equation.

“Estimate the impact of NPIs on the growth in cases when population immunity is at low levels” (line 236), and “assume that repeated imports into the same household are rare in the early stages of the population-level outbreak” (line 248). Was this appropriate given the phase of the epidemic during the analysis period? When does this approach become inappropriate in terms of the epidemic phase? I think that this is an important discussion point.

In our supplementary material we investigate these assumptions by applying the initial conditions formulae derived in Section 2.5 of the main manuscript to cases with a doubling time of three days, starting prevalence 1%, and initial background immunity ranging from zero to 10%. The early growth of the epidemic lines up closely with an exponential growth curve with rate log(2)/3 in all cases, suggesting that calibrating to an exponential growth curve is valid for all of the situations we consider in our case studies, where the maximum starting prevalence is 1% and the maximum starting immunity is 10%.

Line 289 - “In Figure 1 we plot the early growth in cases” - by cases here, do you mean infections?
This is indeed what we meant, and we have corrected “cases” to “infections”.

Lines 354-357. Please be more specific - what was the scaled back policy? And what is the more permissive approach that you explore. I realise that this later becomes more clear but worth stating from the outset of this section.
We have added an explicit definition of the policy which was implemented in December 2020, and have specified that more permissive alternatives would involve either larger bubbles or longer mixing periods (lines 654-668).

Line 360. “We expect that in the scenario we are modelling individuals are likely to mix intensively across risk classes within the household and will mostly not attend school or work, where ordinarily mixing is likely to be highly age-stratified.” Why? Because it’s over the Christmas period? What other NPIs were in place over this period?

Line 362. "This motivates our approximation that stratification by class is not required to capture transmission within a short-term bubble, and thereby keep the dimension of the system manageable" - I think that it would useful to include a brief discussion of if/how this might limit the applicability of your approach to other policy contexts in the discussion.

We have addressed both of points by rewriting our explanation of the choice to use a single age class by emphasising the need for a small system size. Our model scales badly with the number of risk classes in a household, which in a “bubbled” population will be the product of the number of age classes and the number of households per bubble. The explanation of this choice appears on lines 679-684, and we return to it in the Discussion section when we discuss the potential for further work on the computational limits of our model, and how this work could allow us to model more complex risk structures.

Near line 377 - “original NPIs are reimplemented…”. What are these?

We have rephrased our description of the period after the bubbling period as follows: “After the bubbling period, full lockdown measures are reimplemented, with mixing between distinct households forbidden. To simulate the infectious disease dynamics following the bubbling period…” (722-723)

Near line 380, first sentence of section 2.6.3 - “…giving us a SEPIR structure”. Should this be SEPIRQ?

We have corrected this accordingly.

And in the following sentence, “temporarily remove an individual” - is this clearer if revised to “…temporarily remove a (detected) infected individual…”?

We have revised this as you have suggested.

Lines 382-385 - what is meant by the “within-household isolation policy” mentioned here - do the other members of the household quarantine because of the detection of an infection? Is that how between-household transmission is reduced? Wouldn’t the members of that household quarantine even under a policy of OOHI and so both policies reduce between household transmission but the OOHI also reduces within household transmission? Or do you assume individuals isolating within a household are

able to reduce the probability of transmission to other household members? Please clarify the policies and thinking here.

And more broadly, are within-household isolation and contact quarantine accounted for in the policy analyses? Or were these policies not in place over the analysis period.
Thanks for raising this, the mention of within-household isolation in our original manuscript was slightly obscure. By “within-household isolation” we refer to any policy that seeks to control transmission by asking individuals to isolate within their own home on testing positive for COVID-19. In our model we can simulate a within-household isolation policy by reducing the intensity of between-household social contacts for detected infectious individuals. From the perspective of a susceptible individual, this will manifest as a reduction in the amount of between-household infection they are supposed to. On the population level, this in turn manifests as a reduction in the epidemic growth rate. Because we calibrate the between-household transmission in our model to the growth rate during periods where within-household isolation policies were in place, we are implicitly accounting for their impact in our estimation of the transmission parameter beta_ext. If we were also to implement an explicit simulation of within-household isolation, we would effectively incorporate the impact of this policy twice over, once in the fit to growth rate and once in the policy simulation itself. In the specific case of OOHI, our calibration thus implicitly places our simulations in a setting where infectious individuals who do not isolate outside of the household are isolating within their own homes. This raises a more general point about our calibration approach: when we calibrate to SITP and growth rate estimates, we are effectively fitting to the background of policy measures which were in place when those estimates were taken, and so any policy simulations we perform are assumed to be implemented against that background of policies.

This idea was briefly mentioned in the original manuscript on lines 391-395. We have expanded this discussion for increased clarity (lines 800-812).

Results and discussion

Figure 3 caption - the caption says “between-household contact rates” - should these be transmission rates?
We have revised the caption to refer to transmission rates. We emphasise though that since our model assumes transmission rates are linearly proportional to contact rates, these analyses are agnostic as to whether the percentage reduction acts by reducing the number of contacts made by each individual, or reducing the intensity of transmission across each of these contacts.

Line 467 - “the object of such a policy is chiefly to ameliorate the impact of NPIs…and to lessen the psychological impact of isolation on older people living alone” - please amend to isolation of people living alone. The impact of isolation applies to all single person households and you have modelled it as such.
This sentence has been revised in line with the reviewer’s suggestion.

Figures 6 and 7 - please be consistent in figure labels and caption descriptions. Currently plot labelled with single household and bubbled density exponents and caption describes pre-bubbling and during-bubbling density exponents.
We have rephrased the caption so that it reads “... as a function of density exponents in single households and in bubbled households…”.

Line 570-572 - “In practice, high quarantine probabilities…” - should this be isolation probabilities? Otherwise, I’m a little confused. And again lines 586-587 - “while external quarantine” - should this be isolation?
We have corrected both of these appearances of “quarantine” to “isolation”.
